# Clinical Outcomes of Periodontal Surgery Following Crown Reduction for Malocclusion-Associated Gingival Recession in Cats

**DOI:** 10.3390/vetsci13090896

**Published:** 2026-08-31

**Authors:** Kue Hwan Choe, Gyumin Kim, Seong Soo Kang, Se Eun Kim, Hyun Min Jo

**Affiliations:** 1 Taeil Animal Dental Clinics, Seoul 01394, Republic of Korea; taeilah0075@gmail.com; 2 Department of Veterinary Surgery, College of Veterinary Medicine and BK21 Plus Project Team, Chonnam National University, Gwangju 61186, Republic of Korea; vetkang@chonnam.ac.kr (S.S.K.); ksevet@gmail.com (S.E.K.); 3 Jidongbeom Animal Ophthalmologic and Dental Hospital, Busan 46269, Republic of Korea; vetkgm@naver.com; 4 Biomaterial R&BD Center, Chonnam National University, Gwangju 61186, Republic of Korea

**Keywords:** malocclusion, gingival recession, crown reduction, periodontal surgery, cat

## Abstract

Gingival recession from traumatic occlusal contact commonly affects feline mandibular premolars and molars. Although crown reduction is routinely performed to eliminate traumatic occlusal forces, its ability to restore established periodontal defects remains unclear. This study evaluated whether adjunctive periodontal surgery provides additional clinical benefits following crown reduction. This retrospective study included 17 cats (34 teeth) with malocclusion-associated gingival recession. Treatment grouping was performed at the tooth level, with one cat contributing teeth to both the Control and Group A. Teeth were classified into three groups: no treatment (Control), crown reduction alone (Group A), and crown reduction with adjunctive periodontal surgery (Group B). Gingival recession, clinical attachment level (CAL), root coverage, and the vertical bone defect ratio (VBDR) were evaluated before and after treatment. Between-group differences were analyzed using linear mixed-effects models. Control showed no significant changes. Both treatment groups showed significant improvements in gingival recession, CAL, and VBDR. Compared with crown reduction alone, adjunctive surgery yielded significantly greater CAL improvement, radiographic bone healing, and overall clinical success rate (91.7% in Group B vs. 25.0% in Group A), though root coverage did not differ significantly. Traumatic occlusion elimination is foundational for managing feline malocclusion-associated gingival recession. However, adjunctive periodontal surgery enhances periodontal recovery and overall success, offering a valuable tooth-preserving option when complete reconstruction is required.

## Simple Summary

Gingival recession is a common dental problem in cats and can be caused by abnormal contact between the upper and lower teeth. The standard treatment is to reshape the offending tooth to remove this contact, but it is not known whether this alone is enough to restore damaged gum and supporting bone. In this study, we compared three approaches: no treatment, tooth reshaping alone, and tooth reshaping combined with periodontal surgery. Cats treated with tooth reshaping and periodontal surgery showed greater improvement in gum attachment, bone healing, and overall treatment success than cats treated with tooth reshaping alone. These findings suggest that correcting abnormal tooth contact is the first step in treatment, while periodontal surgery may provide additional benefits for cats with more advanced tissue damage. This study provides evidence that preserving affected teeth through periodontal surgery may be a useful treatment option in selected cats and may help guide future clinical decision-making in veterinary dentistry.

## 1. Introduction

Gingival recession refers to the apical migration of the gingival margin from the cementoenamel junction (CEJ) toward the root apex [1]. As gingival recession progresses, destruction of the periodontal supporting tissues, including the gingival connective tissue attachment and alveolar bone, results in root surface exposure and loss of periodontal attachment [2]. Persistent gingival recession and root exposure may lead to increased root sensitivity, plaque and calculus accumulation on the root surface, and inflammatory responses. In cats, gingival recession affecting the mandibular premolar and molar teeth can be broadly classified into two main etiologic categories: recession associated with malocclusion and recession secondary to periodontal disease [3,4].

Malocclusion-associated gingival recession, sometimes described as a gingival cleft, develops when the cusps of the maxillary premolar or molar teeth repeatedly contact the attached gingiva or alveolar mucosa adjacent to the mandibular premolar and molar teeth [5,6]. A study reported that among 44 malocclusion-related lesions in 27 cats, gingival clefts occurred in 25% (11 cases) [3]. Treatment for this problem involves occlusal correction through the contouring of the maxillary premolar cusps or, in severe cases, extraction of the offending teeth [3,5,6]. Although these procedures remove the underlying mechanical cause, it remains unclear whether correction of occlusal trauma alone is sufficient to restore established gingival recession and associated periodontal attachment or alveolar bone loss.

In human dentistry, periodontal surgical approaches have long been established for the management of gingival recession. Commonly used procedures include coronally positioned flap (CPF) and open flap debridement (OFD), which may be combined with regenerative materials such as enamel matrix derivative (EMD) (e.g., Emdogain) or bone grafts to improve periodontal healing [7,8,9,10,11,12,13]. CPF is a surgical technique in which a gingival flap is repositioned coronally to cover the exposed root surface at sites of gingival recession. In human dentistry, CPF is widely used for aesthetic improvement, reduction in dentin hypersensitivity, and facilitation of plaque control [13]. OFD provides surgical access for root surface debridement and management of periodontal defects [7]. These approaches provide a potential rationale for treating established soft-tissue and osseous defects after elimination of traumatic occlusal contact.

Periodontal surgical approaches used in veterinary dentistry have increasingly been adopted from human dentistry as conservative approaches for preserving strategically important teeth. However, their clinical application has largely been limited to the management of periodontal disease, particularly in dogs with vertical periodontal defects, where regenerative procedures such as guided tissue regeneration (GTR) and EMD application have shown favorable clinical outcomes [14,15]. However, reports describing periodontal surgical treatment of malocclusion-associated gingival recession in cats remain scarce, especially in the premolar and molar region. Consequently, the additional benefit of periodontal surgery after correction of traumatic occlusal contact has not been established in feline patients.

Therefore, the present study aimed to compare the clinical outcomes of three management strategies for malocclusion-associated gingival recession affecting the mandibular premolar and molar teeth in cats: no treatment, crown reduction alone, and crown reduction combined with periodontal surgery. The study specifically evaluated whether elimination of traumatic occlusal contact by crown reduction was sufficient to promote periodontal recovery and whether adjunctive periodontal surgery provided additional clinical benefits. To the best of the authors’ knowledge, this is the first study to directly compare untreated lesions, crown reduction alone, and crown reduction combined with periodontal surgery for feline malocclusion-associated gingival recession.

## 2. Materials and Methods

### 2.1. Case Inclusion

A total of 17 cats (34 teeth) diagnosed with malocclusion-associated gingival recession affecting the mandibular premolar and molar teeth were retrospectively included in this study. Only cats with adequate oral hygiene and whose owners were able to perform home brushing were included, irrespective of the treatment performed. Inclusion criteria were limited to cases in which at least one follow-up examination under anesthesia was conducted within 1–4 years postoperatively. During the follow-up period, periodontal pocket depth and dental radiographs were evaluated, and pre- and post-treatment values were compared. Clinical patient data, including age, sex, breed, body weight, treated teeth, application of EMD, and the presence of other oral diseases, were recorded in a periodontal examination chart.

Treatment decisions were made during routine clinical care after discussion with the owners regarding the available treatment options and associated costs. Teeth were subsequently classified into three groups according to the treatment performed. One cat contributed teeth to both the Control and Group A, whereas all other cats contributed teeth to a single treatment group. The control group included teeth that received no treatment during the observation period. Group A included teeth treated with crown reduction alone, whereas Group B included teeth treated with crown reduction combined with periodontal surgery.

### 2.2. Surgical Procedures

#### 2.2.1. Anesthesia

For general anesthesia, the following pre-anesthetic agents were administered: Ampicillin (Penbrex inj; Yungjin Pharm, Seoul, Republic of Korea) at a dosage of 20 mg/kg, butorphanol (Butophan; Myungmoon Pharm, Seoul, Republic of Korea) at 0.1 mg/kg, and midazolam (Bukwang Midazolam; Bukwang Pharm, Seoul, Republic of Korea) at 0.2 mg/kg. Anesthesia was initiated with intravenous injection of propofol (1%) (Anepol; Hana Pharm, Seongnam-si, Republic of Korea) at a dosage of 4 mg/kg. Anesthesia was maintained with isoflurane subsequent to endotracheal intubation. Before dental treatment, all cats received regional nerve blocks using 0.5% bupivacaine hydrochloride (Myungmoon Bupivacaine Hydrochloride Injection; Myungmoon Pharm, Seoul, Republic of Korea) at a total dose of 1 mg/kg for perioperative analgesia.

#### 2.2.2. Crown Reduction

In groups A and B, the gingival cleft was identified, and the contacting portion of the offending maxillary cusp responsible for the traumatic contact was selectively reduced using a high-speed handpiece with a fine-grit flame-shaped diamond bur under copious water irrigation. In all cases, crown reduction was limited to approximately 0.3–0.5 mm to avoid pulp exposure. The reduced surface was finished with Sof-Lex™ Polishing Discs (3M ESPE, St. Paul, MN, USA) and polished using OneGloss^®^ PS (Shofu Inc., Kyoto, Japan). The enamel surface was then etched with 37% phosphoric acid etching gel, rinsed with water, and air-dried. A universal adhesive (Prime&Bond Universal, Dentsply Sirona, Konstanz, Germany) was applied and light-cured, followed by application of a resin surface sealant (Fortify^®^, Bisco Inc., Schaumburg, IL, USA) and light curing according to the manufacturer’s instructions.

#### 2.2.3. Periodontal Surgery

In Group B, teeth underwent OFD in combination with EMD (Emdogain^®^, Straumann, Basel, Switzerland) application. Additionally, a CPF was performed to reposition the recessed gingiva to its normal position. A gingival envelope incision was made in the mandibular premolar and molar region, followed by full-thickness flap elevation. The exposed root surface underwent root planing and debridement, after which EMD was applied to the root surface. A periosteal releasing incision was then performed to achieve tension-free coronal advancement of the flap, and the surgical site was sutured with 5-0 poliglecaprone 25 (Monocryl^®^, Ethicon, Somerville, NJ, USA).

### 2.3. Postoperative Management

All cats received standardized postoperative care. Antimicrobial therapy consisted of a single subcutaneous injection of cefovecin (8 mg/kg; Convenia, Zoetis, Parsippany, NJ, USA). Analgesic therapy included gabapentin (5 mg/kg orally twice daily for 14 days; Neurontin Cap. 100 mg, Viatris Korea, Seoul, Republic of Korea).

Follow-up examinations were conducted at 2 weeks, 1 month, and 2 months postoperatively for all cases. Thereafter, follow-up intervals were individualized according to each patient’s oral hygiene status and owner compliance, ranging from 1 to 6 months. Throughout the follow-up period, intraoral photographs were obtained at each visit to assess and document the adequacy of home care and oral hygiene management.

### 2.4. Outcome Measurements

The surgical outcomes were assessed under anesthesia after at least one year postoperatively. To determine the success of periodontal surgery, both CAL assessments using a periodontal probe under anesthesia and radiographic evaluation of VBDR were performed [14]. A site was deemed a clinical success if both VBDR and CAL satisfied the predetermined success criteria when evaluating pre- and post-treatment records.

#### 2.4.1. Clinical Attachment Level (CAL) Assessment

**Figure 1:**
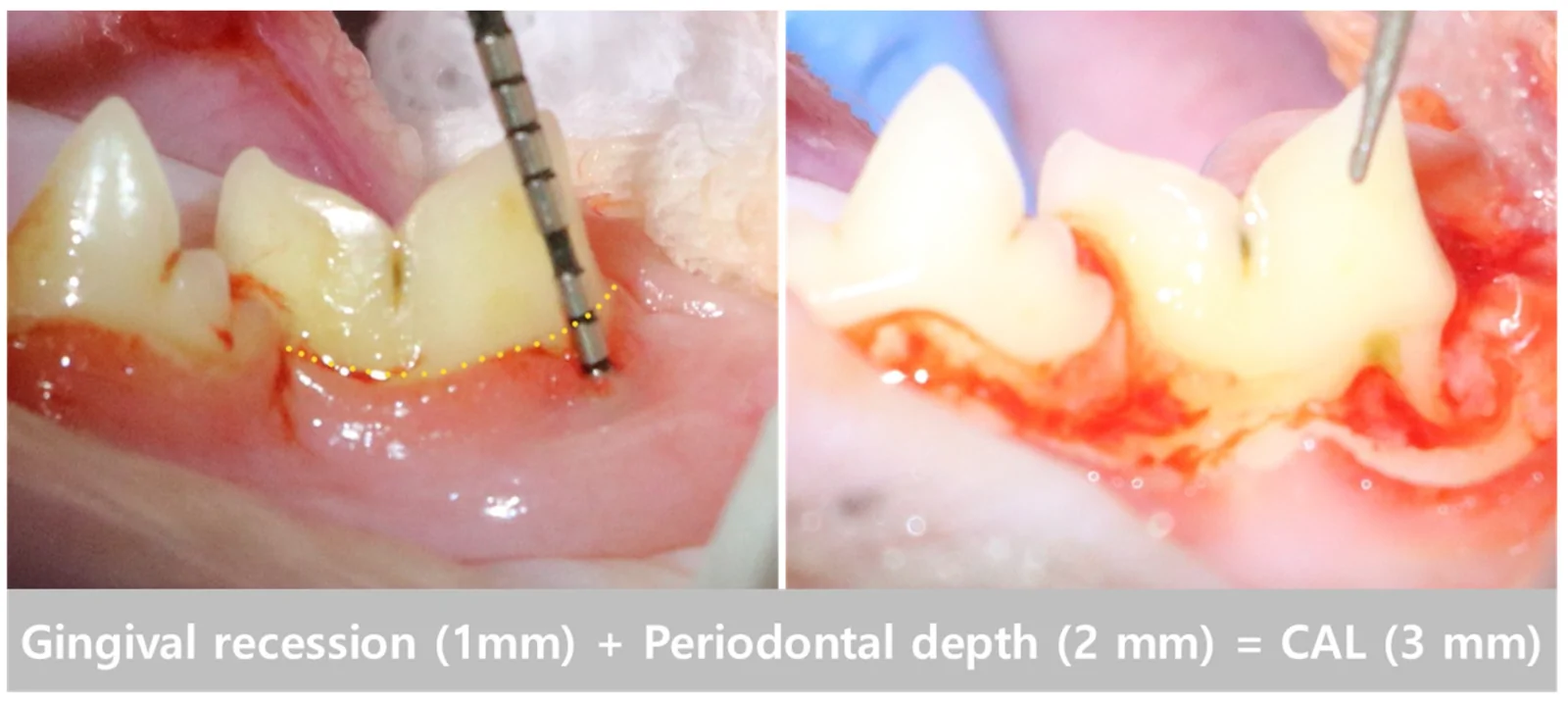
Clinical attachment level (CAL) measurement. CAL accounts for both gingival recession and periodontal depth (PD). The level of the cementoenamel junction is demarcated by the dashed line (yellow).

The CAL was assessed by measuring probing depth (PD) and the degree of gingival recession with a periodontal probe. A reduction in CAL by at least 1 mm compared to the preoperative value was considered successful treatment (Figure 1).

#### 2.4.2. Vertical Bone Defect Ratio (VBDR) Assessment

**Figure 2:**
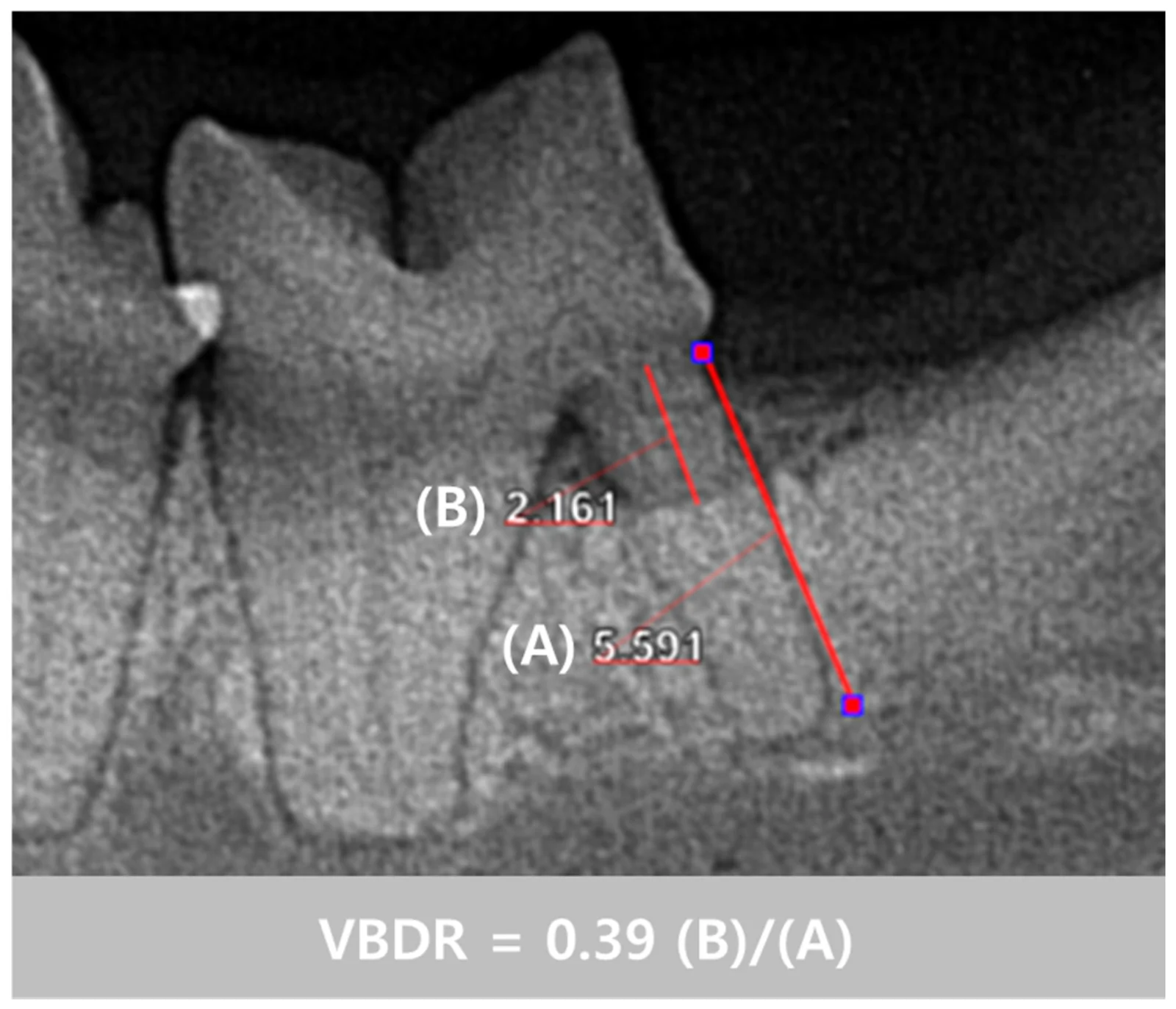
Vertical bone defect ratio (VBDR). Measurements were performed using the line tool to draw lines (red lines) to measure both (A) root length, defined as the distance from the cementoenamel junction to the apex, and (B) the vertical bone pocket depth, defined as the distance from the cementoenamel junction to the most apical aspect of bone loss. The bone pocket depth ratio was generated as the ratio of the vertical bone pocket depth to the root length.

The vertical bone defect ratio (VBDR) was measured using dental radiographs obtained under anesthesia by a single examiner blinded to the treatment group. The preoperative and postoperative values (after at least one year) were compared to assess treatment success. A reduction in VBDR to less than 90% of the preoperative value was classified as improvement. A postoperative VBDR within ±10% of the preoperative value was classified as maintenance. An increase in VBDR exceeding 110% of the preoperative value was classified as deterioration (Figure 2.)

#### 2.4.3. Root Coverage Assessment

Root coverage was evaluated based on changes in gingival recession measurements. The amount of root coverage was calculated as the difference in gingival recession between pre- and post-operative measurements.

### 2.5. Statistical Analysis

Statistical analyses were performed using jamovi (version 2.7.17) and R statistical software (version 4.5.2; R Foundation for Statistical Computing, Vienna, Austria) within the RStudio (version 2026.01.0+392; Posit Software, PBC, Boston, MA, USA) integrated development environment. Data distribution was assessed visually and using descriptive statistics. As most variables did not meet the assumption of normality, results are presented as median with interquartile range (IQR).

Within-group pre- and post-treatment comparisons were conducted using paired non-parametric tests. Between-group differences in changes in gingival recession (ΔGR), root coverage, clinical attachment level (ΔCAL), and vertical bone defect ratio (ΔVBDR) were evaluated using linear mixed-effects models (LMMs), with treatment group included as a fixed effect and cat ID included as a random intercept to account for repeated measurements within individual cats. Model parameters were estimated using restricted maximum likelihood (REML), and degrees of freedom were calculated using the Satterthwaite approximation.

Data visualization was performed using R. Statistical significance was set at *p* < 0.05.

## 3. Results

### 3.1. Study Population

Baseline characteristics of the cats and teeth in each treatment group are summarized in Table 1.

#### 3.1.1. Control Group

The control group comprised five cats with 10 teeth affected by malocclusion-associated gingival recession. The mean age was 6.8 years (range, 4–10 years). The group included two castrated males and three spayed females. The mean body weight was 4.9 kg (range, 4.0–6.0 kg), and the mean follow-up duration was 28.0 months (range, 12–36 months).

#### 3.1.2. Group A: Crown Reduction

Group A comprised six cats with 12 teeth affected by malocclusion-associated gingival recession. The mean age was 4.8 years (range, 1–9 years). The group included five castrated males and one spayed female. The mean body weight was 5.4 kg (range, 4.6–6.3 kg), and the mean follow-up duration was 28.0 months (range, 12–48 months).

#### 3.1.3. Group B: Crown Reduction Combined with Periodontal Surgery

Group B comprised seven cats with 12 teeth affected by malocclusion-associated gingival recession. The mean age was 3.4 years (range, 2–9 years). The group included three castrated males and four spayed females. The mean body weight was 4.8 kg (range, 3.5–6.0 kg), and the mean follow-up duration was 24.0 months (range, 12–48 months).

### 3.2. Representative Clinical and Radiographic Findings

**Figure 3:**
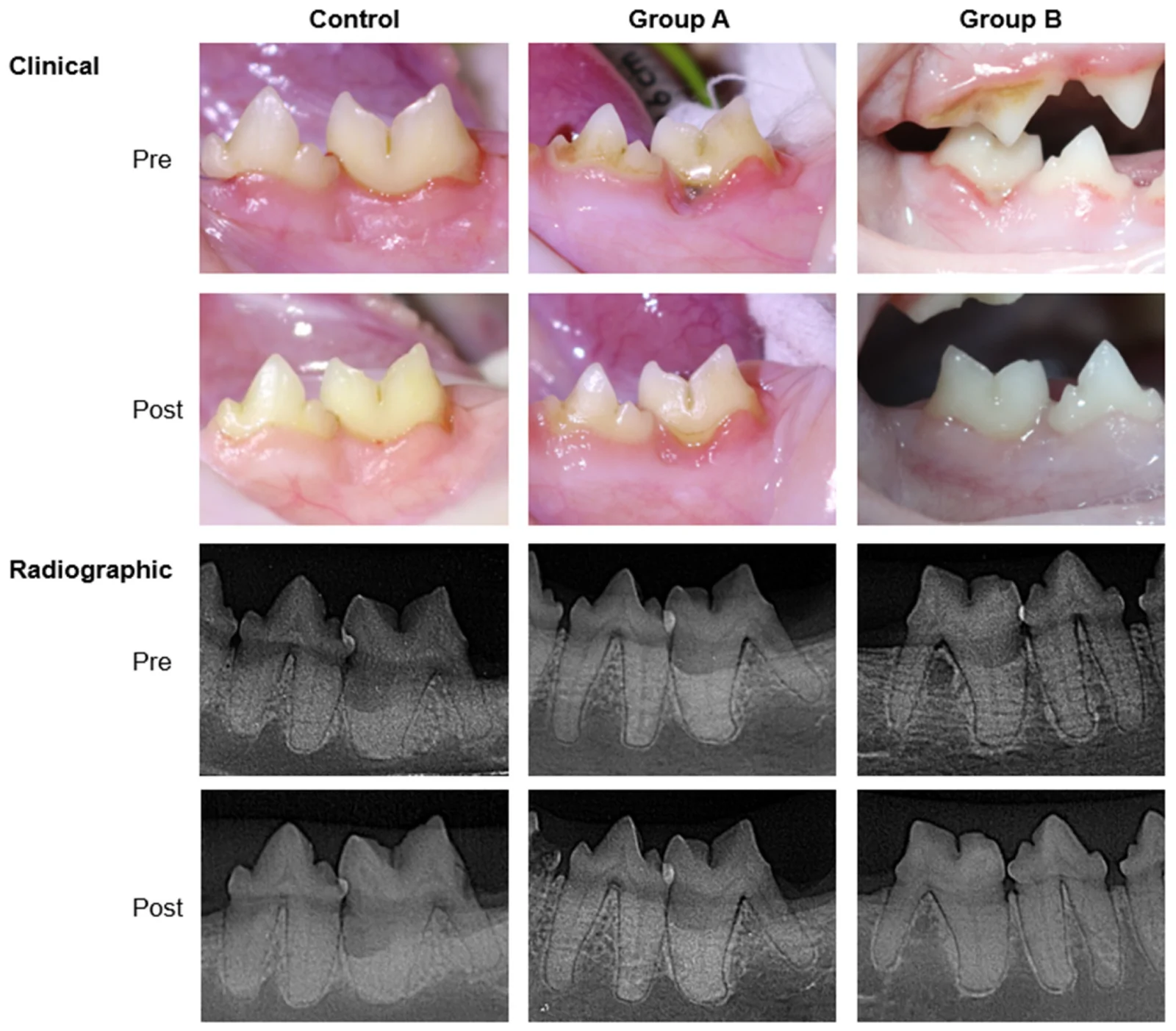
Representative clinical photographs and intraoral radiographs obtained before (Pre) and after (Post) treatment in each study group. The Control group received no treatment. Group A underwent crown reduction alone, whereas Group B underwent crown reduction followed by periodontal surgery (OFD, CPF, and EMD application).

Representative clinical photographs and intraoral radiographs from each treatment group are presented in Figure 3. The Control group showed little change in gingival recession or alveolar bone loss during follow-up. Group A demonstrated improvement in gingival recession following crown reduction, whereas Group B exhibited more pronounced improvement in both soft-tissue coverage and radiographic bone healing after adjunctive periodontal surgery.

### 3.3. Within-Group Clinical Changes After Surgery

**Figure 4:**
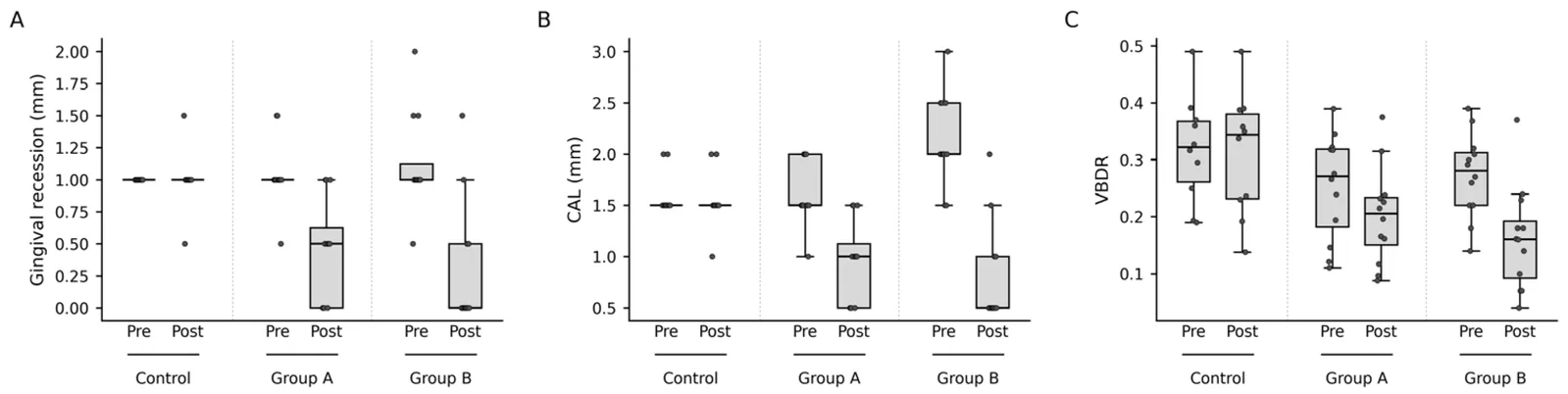
Changes in clinical and radiographic parameters before and after surgery. Changes in gingival recession (**A**), clinical attachment level (CAL) (**B**), and vertical bone defect ratio (VBDR) (**C**) before and after treatment in the Control, Group A, and Group B. Box plots represent the median and interquartile range (IQR), and individual dots indicate site-level measurements.

Within-group comparisons revealed no significant changes in gingival recession, clinical attachment level (CAL), or vertical bone defect ratio (VBDR) in the control group following the observation period (Table 2, Figure 4A–C).

In contrast, significant improvements were observed across all clinical parameters in Group A. The median gingival recession decreased from 1.000 mm preoperatively to 0.500 mm postoperatively (*p* = 0.002). Likewise, the median CAL decreased from 1.500 mm before treatment to 1.000 mm after treatment (*p* = 0.002). The median VBDR also decreased significantly from 0.271 preoperatively to 0.206 postoperatively (*p* < 0.001), indicating radiographic improvement in vertical bone defects.

Similarly, Group B demonstrated significant improvements in all evaluated parameters. The median gingival recession decreased from 1.000 mm preoperatively to 0.000 mm postoperatively (*p* < 0.001). The median CAL decreased from 2.000 mm before treatment to 0.500 mm after treatment (*p* < 0.001), while the median VBDR decreased from 0.281 preoperatively to 0.161 postoperatively (*p* < 0.001), indicating significant radiographic improvement in vertical bone defects.

### 3.4. Between-Group Comparison of Treatment Effects

**Figure 5:**
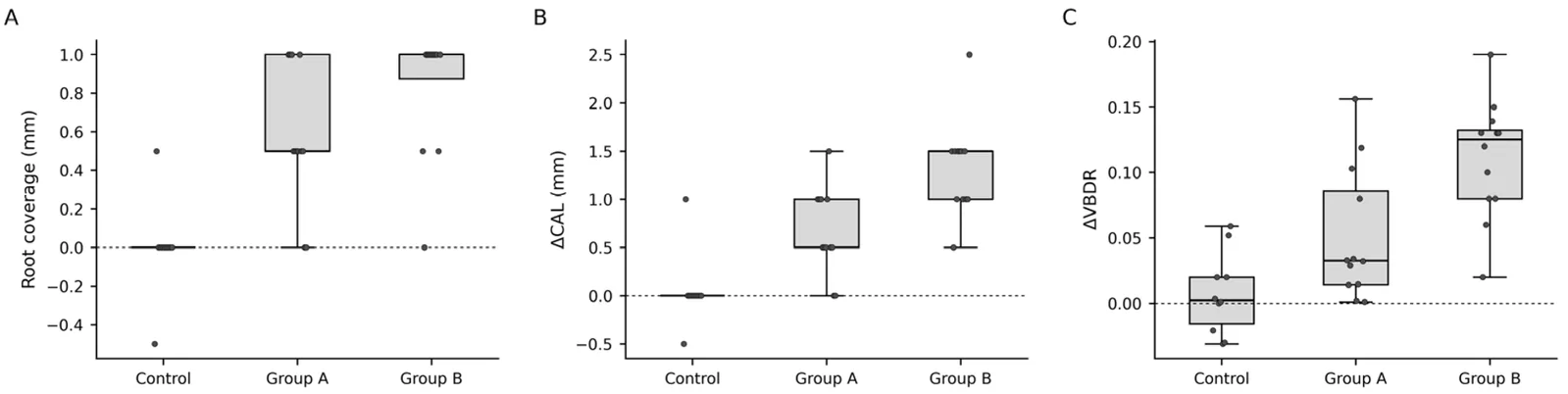
Between-group comparisons of changes in clinical and radiographic parameters. Comparisons of changes in root coverage (**A**), clinical attachment level (ΔCAL) (**B**), and vertical bone defect ratio (ΔVBDR) (**C**) among the Control, Group A, and Group B. Changes were calculated as pre-treatment minus post-treatment values, with positive values indicating improvement. Box plots represent the median and interquartile range (IQR), and individual dots indicate site-level measurements.

Between-group comparisons using linear mixed-effects models revealed significant differences in treatment-related changes between the study groups (Table 3, Figure 5).

For root coverage, both Group A and Group B showed significantly greater improvements than the Control group (Control vs. Group A: estimate, 0.531 ± 0.161 mm; 95% CI, 0.202–0.861; *p* = 0.005; Control vs. Group B: estimate, 0.742 ± 0.185 mm; 95% CI, 0.348–1.137; *p* = 0.003). However, no significant difference was observed between Group A and Group B (estimate, 0.211 ± 0.176 mm; 95% CI, −0.167–0.589; *p* = 0.251).

For ΔCAL, Group A and Group B both demonstrated significantly greater improvements than the Control group (Control vs. Group A: estimate, 0.467 ± 0.202 mm; 95% CI, 0.055–0.878; *p* = 0.028; Control vs. Group B: estimate, 1.273 ± 0.263 mm; 95% CI, 0.718–1.829; *p* < 0.001). In addition, the improvement in ΔCAL was significantly greater in Group B than in Group A (estimate, 0.807 ± 0.253 mm; 95% CI, 0.269–1.345; *p* = 0.012).

For ΔVBDR, Group B showed significantly greater improvement than both the Control group (estimate, 0.101 ± 0.021; 95% CI, 0.053–0.149; *p* = 0.003) and Group A (estimate, 0.059 ± 0.020; 95% CI, 0.014–0.104; *p* = 0.032). No significant difference was observed between the Control group and Group A (estimate, 0.042 ± 0.021; 95% CI, −0.004–0.088; *p* = 0.068).

### 3.5. Clinical Success Rates

Clinical success rates based on the predefined CAL, VBDR, and combined success criteria are summarized in Table 4. In the Control group, CAL success was achieved in 1 of 10 sites (10.0%), while VBDR success was observed in 2 of 10 sites (20.0%). None of the sites fulfilled the predefined combined success criterion. In Group A, CAL success was achieved in 5 of 12 sites (41.7%), whereas VBDR success was observed in 9 of 12 sites (75.0%). However, only 3 of 12 sites (25.0%) fulfilled both criteria and were classified as combined clinical successes. In Group B, CAL success, VBDR success, and combined clinical success were each achieved in 11 of 12 sites (91.7%), representing the highest success rates among the three groups (Table 4).

## 4. Discussion

In human dentistry, CPF procedures are well-established surgical techniques [8]; however, several factors limit their application in feline patients, underscoring the need for meticulous surgical planning and execution. Consequently, despite the availability of various periodontal surgical procedures and regenerative materials, such interventions have rarely been reported in cats.

One important limiting factor relates to the anatomical characteristics of feline oral tissues. Cats possess relatively small teeth and delicate gingival tissues, which are more fragile than those of dogs, making them susceptible to trauma during flap elevation and suturing [16]. Another important consideration involves functional and structural aspects of feline occlusion. Cats maintain dental cleanliness in part through lateral occlusal movements [17], and the narrow interdental spaces, combined with factors such as foreign body sensation, dental pathology, or aggressive tooth brushing, may result in repeated contact between the cusps of the maxillary premolar teeth and the gingiva or oral mucosa of the mandibular premolar and molar teeth. Consequently, careful assessment of occlusal relationships and the risk of maxillary cusp-induced trauma is essential before undertaking periodontal surgery in feline mandibular teeth. Collectively, these factors may adversely affect surgical outcomes and overall prognosis.

This study evaluated the clinical outcomes of several treatment strategies for malocclusion-associated gingival recession affecting feline mandibular premolar and molar teeth, comparing an untreated control group, coronal reduction alone, and coronal reduction combined with a periodontal surgical approach consisting of OFD, CPF, and EMD application. Both treatment strategies significantly improved gingival recession, CAL, and VBDR, whereas no significant changes were observed in the untreated control group. Although coronal reduction alone produced favorable clinical outcomes, adjunctive periodontal surgery resulted in significantly greater improvements in both clinical attachment level and radiographic bone healing, together with a substantially higher overall clinical success rate. These findings suggest that elimination of traumatic occlusal forces is a key component in the management of malocclusion-associated gingival recession, while adjunctive periodontal surgery may provide additional benefits in selected cases.

The superior clinical and radiographic outcomes observed following adjunctive periodontal surgery may be attributed to the complementary effects of OFD, CPF, and EMD. OFD provides access for root debridement and preparation of the recipient site, whereas CPF achieves stable root coverage and minimizes mechanical disruption of the healing wound [18]. In addition, EMD has been shown to promote periodontal wound healing and regeneration by enhancing fibroblast proliferation and suppressing epithelial downgrowth [19,20]. The complementary effects of these procedures may explain the favorable clinical and radiographic outcomes observed in the present study.

Based on these biological mechanisms, the combination of OFD, CPF, and EMD may represent a practical regenerative approach in feline periodontal surgery. Previous research in dogs with surgically created periodontal defects demonstrated that treatment protocols incorporating EMD, either alone or in combination with guided tissue regeneration (GTR), resulted in comparable new cementum formation and were superior to OFD alone [21,22]. Furthermore, the addition of GTR to EMD did not provide additional regenerative benefits beyond those achieved with EMD alone [11]. Although GTR remains a widely used regenerative technique [23], its clinical application can be technically demanding because of the need for membrane stabilization and the risk of membrane-related complications [24,25]. Therefore, EMD-based approaches may offer sufficient regenerative potential without the routine use of barrier membranes, particularly in anatomically constrained regions such as the feline mandibular premolar and molar teeth.

The greater improvements in both clinical attachment and radiographic bone healing observed in Group B are consistent with the proposed biological effects of OFD, CPF, and EMD discussed above. Elimination of traumatic occlusal contact likely created a more favorable environment for periodontal healing, while adjunctive periodontal surgery may have further enhanced hard-tissue recovery through improved wound stability and regenerative stimulation. Nevertheless, radiographic improvement should not be interpreted as definitive evidence of true periodontal regeneration because histologic evaluation was not performed. Therefore, although the reduction in VBDR indicates favorable alveolar bone healing, confirmation of true periodontal regeneration, including the formation of new cementum, periodontal ligament, and alveolar bone, would require histologic investigation.

These findings have several important clinical implications. Although elimination of traumatic occlusal contact should remain the primary treatment objective, the significantly greater improvements in clinical attachment, radiographic bone healing, and overall treatment success rate observed with adjunctive periodontal surgery suggest that it may provide additional benefits in cases requiring more complete periodontal recovery. Although the regenerative potential of the treated sites could not be confirmed histologically, the concurrent improvements in both clinical attachment level and radiographic bone healing support the clinical value of incorporating periodontal surgery into the management of malocclusion-associated gingival recession in selected cases.

This study has several limitations. First, the individual effects of OFD, CPF, and EMD could not be evaluated separately because these procedures were performed in combination. Therefore, the relative contribution of each component to the observed clinical improvement remains unclear. Second, the relatively small sample size may limit the generalizability and statistical power of the findings. As this was a retrospective study, a priori sample size calculation was not performed, and treatment selection was not randomized. In addition, clinical measurements obtained during routine clinical care could not be assessed in a blinded manner. Larger prospective studies are warranted to confirm these findings. Third, although intraoral radiography was used to evaluate alveolar bone healing, more advanced imaging modalities such as cone-beam computed tomography may allow more accurate assessment of periodontal bone changes. Finally, postoperative plaque control and home oral care were not standardized among cases, which may have influenced periodontal healing and long-term treatment outcomes. Accordingly, the present findings should be interpreted as preliminary.

## 5. Conclusions

This study demonstrated that both crown reduction alone and crown reduction combined with adjunctive periodontal surgery improved periodontal outcomes in cats with malocclusion-associated gingival recession affecting the mandibular premolar and molar teeth. Compared with crown reduction alone, adjunctive periodontal surgery resulted in greater improvements in clinical attachment, radiographic bone healing, and overall treatment success. These findings support the use of adjunctive periodontal surgery as a tooth-preserving treatment option in selected feline patients requiring more complete periodontal reconstruction. Further prospective studies with larger case numbers are warranted to establish evidence-based treatment recommendations for the management of malocclusion-associated gingival recession in cats.

## Figures and Tables

**Table 1:** Baseline characteristics of cats and teeth are in each treatment group.

Variable	Control	Group A	Group B
Study population	-	-	-
Number of cats, *n*	5	6	7
Number of teeth, *n*	10	12	12
Age (years), mean (range)	6.8 (4–10)	4.8 (1–9)	3.4 (2–9)
Sex (CM/SF), *n*	2/3	5/1	3/4
Body weight (kg), mean (range)	4.9 (4.0–6.0)	5.4 (4.6–6.3)	4.8 (3.5–6.0)
Follow-up (months), mean (range)	28.0 (12–36)	28.0 (12–48)	24.0 (12–48)

CM; castrated male, SF; spayed female.

**Table 2:** Within-group changes in gingival recession, clinical attachment level (CAL), and vertical bone defect ratio (VBDR) before and after treatment.

Variable	Group	Time	Median (IQR)	*p*-Value
Gingival recession (mm)	Control	Pre	1.000 (0.000)	n/s
		Post	1.000 (0.000)	
	Group A	Pre	1.000 (0.000)	0.002
		Post	0.500 (0.625)	
	Group B	Pre	1.000 (0.125)	<0.001
		Post	0.000 (0.500)	
CAL (mm)	Control	Pre	1.500 (0.000)	n/s
		Post	1.500 (0.000)	
	Group A	Pre	1.500 (0.500)	0.002
		Post	1.000 (0.625)	
	Group B	Pre	2.000 (0.500)	<0.001
		Post	0.500 (0.500)	
VBDR	Control	Pre	0.322 (0.106)	n/s
		Post	0.344 (0.149)	
	Group A	Pre	0.271 (0.137)	<0.001
		Post	0.206 (0.083)	
	Group B	Pre	0.281 (0.092)	<0.001
		Post	0.161 (0.100)	

**Table 3:** Linear mixed-effects model estimates of between-group differences in root coverage, clinical attachment level (ΔCAL), and vertical bone defect ratio (ΔVBDR).

Variable	Comparison	Estimate ± SE	95% CI	*p*-Value
Root coverage (mm)	Control vs. Group A	0.531 ± 0.161	0.202–0.861	0.005
	Control vs. Group B	0.742 ± 0.185	0.348–1.137	0.003
	Group A vs. Group B	0.211 ± 0.176	−0.167–0.589	0.251
ΔCAL (mm)	Control vs. Group A	0.467 ± 0.202	0.055–0.878	0.028
	Control vs. Group B	1.273 ± 0.263	0.718–1.829	<0.001
	Group A vs. Group B	0.807 ± 0.253	0.269–1.345	0.012
ΔVBDR	Control vs. Group A	0.042 ± 0.021	−0.004–0.088	0.068
	Control vs. Group B	0.101 ± 0.021	0.053–0.149	0.003
	Group A vs. Group B	0.059 ± 0.020	0.014–0.104	0.032

**Table 4:** Clinical success rates according to predefined CAL and VBDR success criteria.

Group	*n*	CAL Success	VBDR Success	Combined Success
Control	10	1/10 (10.0%)	2/10 (20.0%)	0/10 (0%)
Group A	12	5/12 (41.7%)	9/12 (75%)	3/12 (25.0%)
Group B	12	11/12 (91.7%)	11/12 (91.7%)	11/12 (91.7%)

Clinical success for CAL was defined as a reduction ≥1.0 mm between pre- and post-operative measurements, and VBDR success was defined as a ≥10% reduction from baseline. Combined success was defined as achieving both CAL success (≥1.0 mm reduction) and VBDR success (≥10% reduction) at the same site.

## Data Availability

All data generated or analyzed during this study are included in this published article. Further inquiries can be directed to the corresponding author.

## Abbreviations

The following abbreviations are used in this manuscript:

CPF	Coronally positioned flap
OFD	Open flap debridement
CAL	Clinical attachment level
VBDR	Vertical bone defect ratio
EMD	Enamel matrix derivative
